# Supplementary material for: The Australasian dingo archetype: De novo chromosome-length genome assembly, DNA methylome, and cranial morphology
Source: bioRxiv. 2023 Jan 27:2023.01.26.525801. Preprint. [Version 1] doi: 10.1101/2023.01.26.525801 (PMC9900879; doi:10.1101/2023.01.26.525801)
Supplement: Supplement 1 [file media-1.pdf]

Additional Files

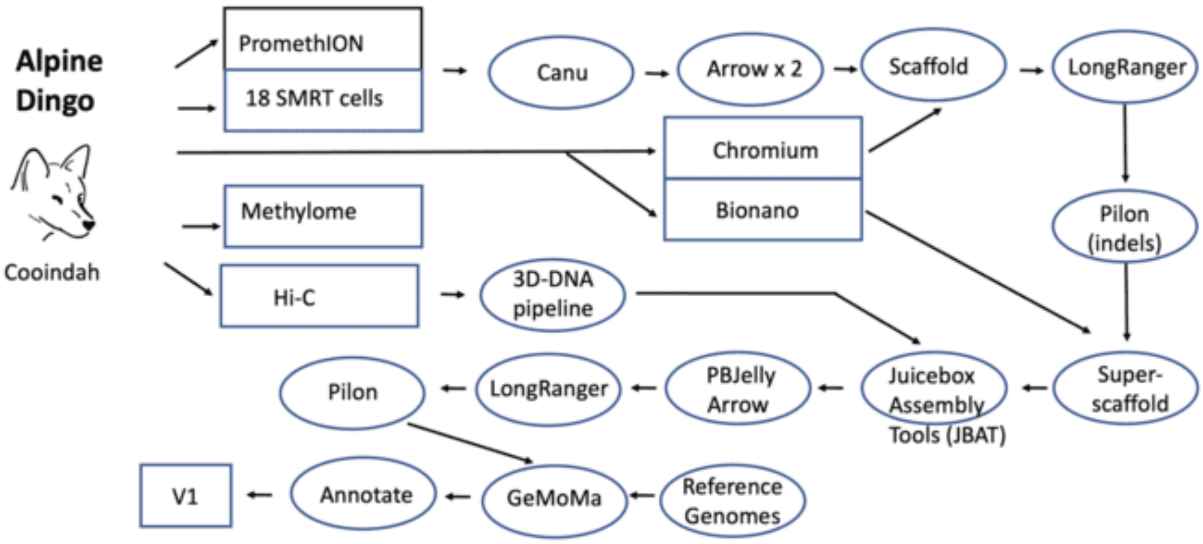

Supplementary Figure 1 Title. Schematic overview of project workflow

Supplementary Figure 1 Legend. Alpine Dingo Cooinda DNA was derived from blood of a single female from the Dingo Sanctuary Bargo. Sequences were generated on the Pacific Biosciences Sequel instrument (V2 chemistry) and Oxford Nanopore PromethION instrument (guppy bascaller Version 3.0.6+9999d81) to ~30x genome coverage, each, based on a genome size estimate of 2.4 Gb (this estimate is used for all coverage estimates). All long read sequences were assembled with the Canu v1.8 algorithm then error corrected twice using the Arrow genomic consensus polishing module. The assembly was scaffolded with Chromium 10x linked-reads (~41x coverage excluding the barcode) using Long Ranger v2.1.6 using DNA from the same animal. Polishing of the assembly for residual indels was done by aligning the Illumina data with Minimap2 and the Racon algorithm. Single molecule Bionano data (~57x effective coverage) was then used to superscaffold the sequence assembly using DNA extracted from the same canid. For this, single molecule optical maps were first de novo assembled into consensus maps, which were than aligned to the sequence assembly in silico digested with the same labelling enzyme for hybrid scaffolding, using Bionano Solve (v3.2.2_08022018) with RefAligner (7782.7865rel). This assembly was

further scaffolded to chromosome-length by DNA Zoo (www.dnazoo.org/methods). Briefly, an *in situ* Hi-C library was prepared from the same individual and sequenced to 29x coverage. The Hi-C data was processed using Juicer [111], and used as input into the 3D-DNA pipeline [112] to produce a candidate chromosome-length genome assembly. We performed additional finishing on the scaffolds using Juicebox Assembly Tools [113]. The assembly was then long-read gap filled with the PBJelly algorithm, and the additional data error corrected using Arrow [32]. The Chromium data was mapped onto the assembly with the Long Ranger v2.1.6 program and the final assembly was then polished using the Pilon algorithm. Of the 2.4 Gb assembled genome, the total assembly N50 contig and scaffold lengths are 23.1 Mb and 64.8 Mb, respectively. The assembled contigs were then aligned to CanFam3.1 for chromosome assignments. Regulatory landscape was characterised by whole genome bisulphite sequencing.

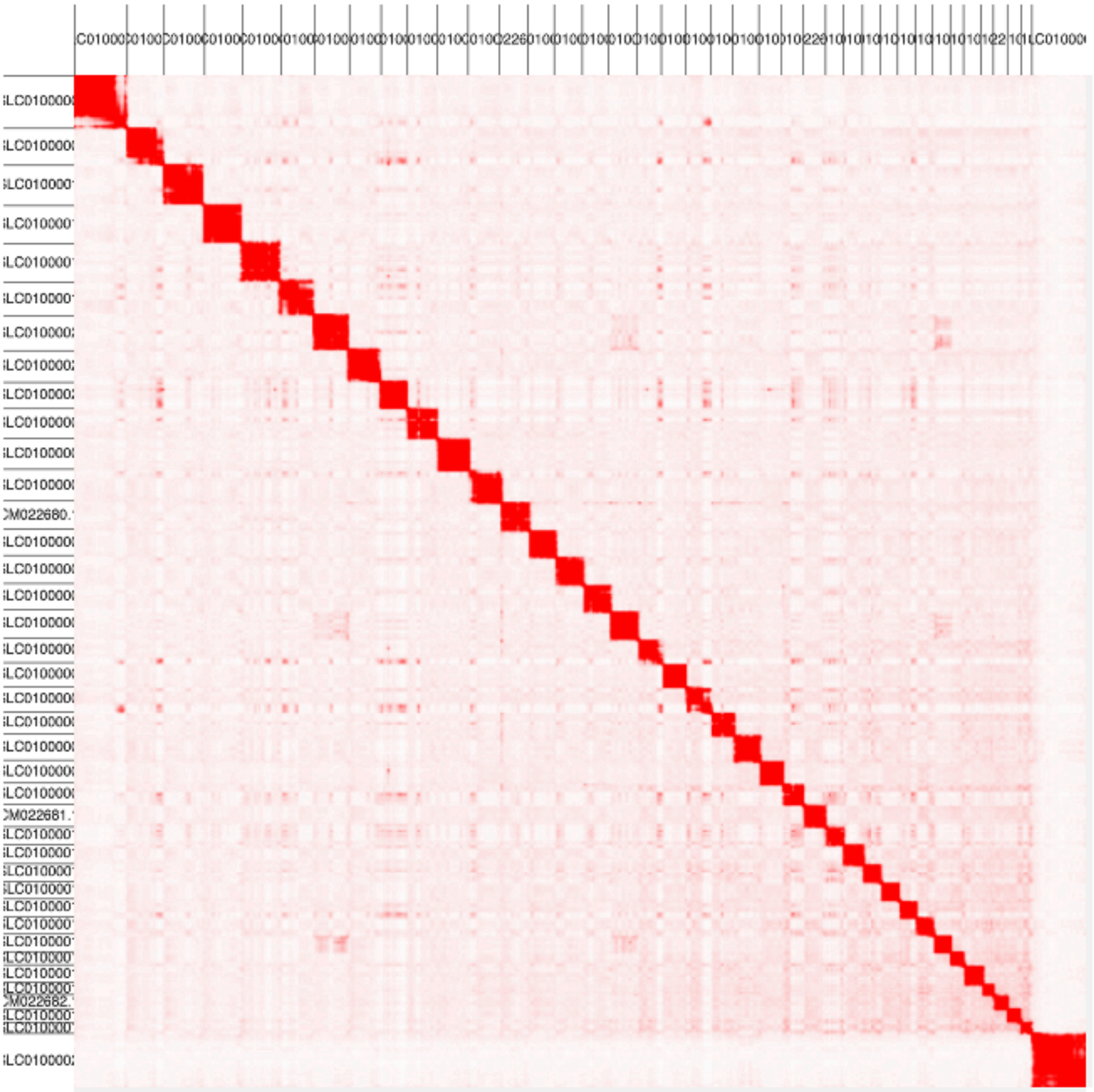

Supplementary Figure 2 Title: Alpine dingo assembly after Hi-C correction

Supplementary Figure 2 Legend: Contact matrices (visualised in Juicebox.js) after the chromosome-length Hi-C upgrade. The chromosome-length contact map can be viewed at multiple resolutions using Juicebox.js [34] following the link https://tinyurl.com/ycbkezf4.

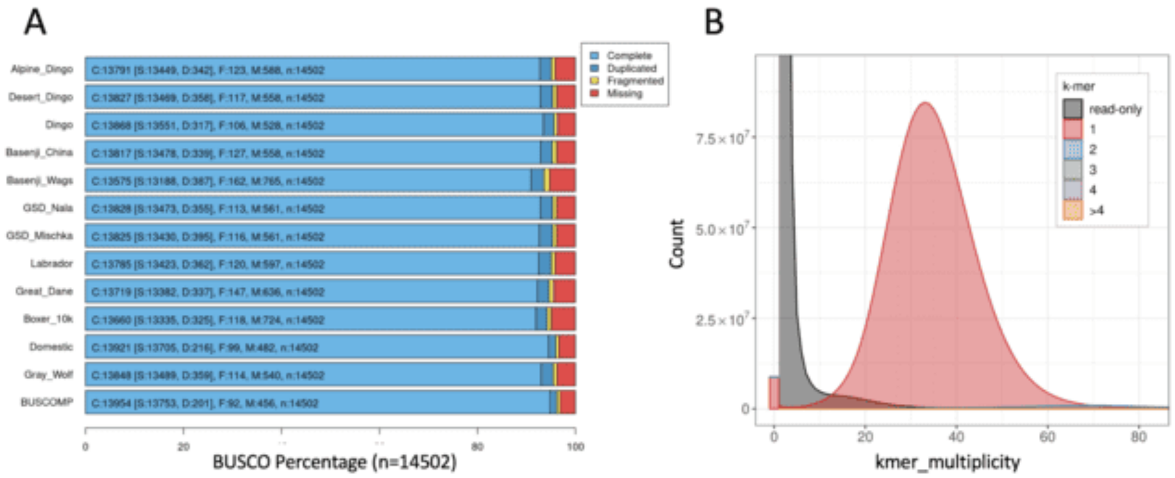

Supplementary Figure 3 Title: Assembly statistics

Supplementary Figure 3 Legend: (A) BUSCO ratings for Cooinda assembly, compared to CanFam4. Purple, original assembly; Black, scaffolding/polishing steps; Blue, final assembly; Red, CanFam4. Dashed red lines mark CanFam4 statistics.

(B) 10x read kmers frequency distributions for kmers with different assembly copy numbers derived from A Read 1 (16bp barcodes trimmed) and B Read 2 (barcodes not trimmed).

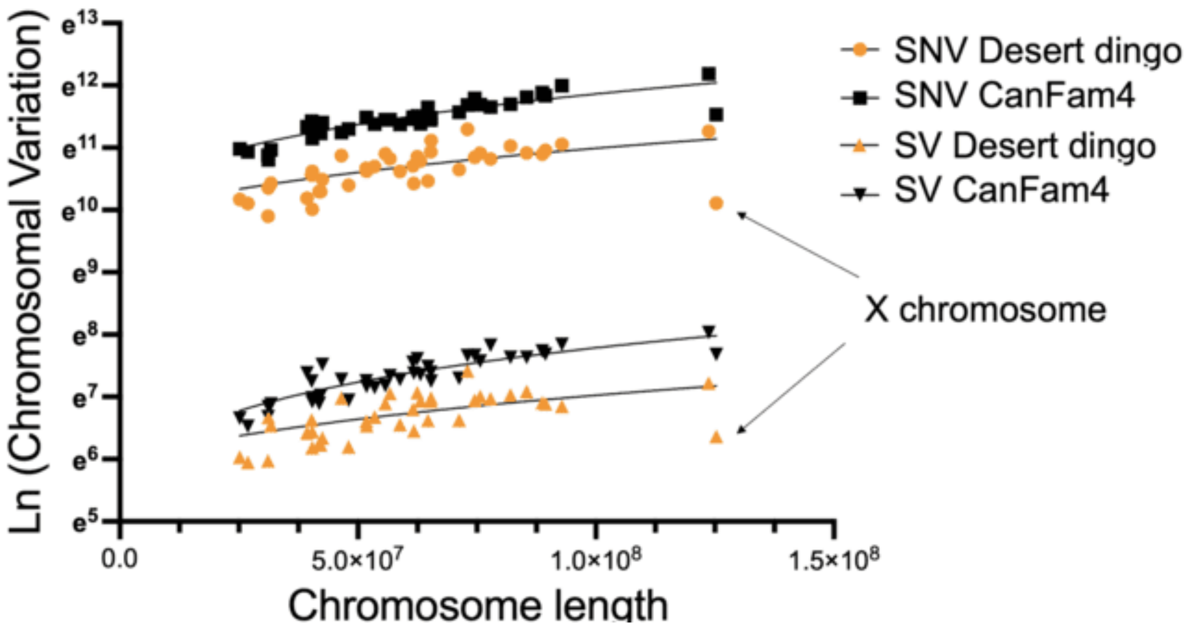

Supplementary Figure 4: Genome shows a deficiency of variation on the X chromosome

Supplementary Figure 4 Legend: SNV (single nucleotide variant) and SV (structural variation) comparisons show a relative deficiency of variation on the X chromosome. Line represents a regression through the non-transformed data and each point represents one chromosome with the length of the Alpine dingo and SNV's or SV relative to the Desert dingo genome or CanFam4. Y=3.8e-4x+21305, 1.1e-4+31753, 7.2e-5+406.7, 2.5e-5+363.1 with an r^2 of 0.37, 0.74, 0.33, 0.77 for SNV Desert dingo, SNV CanFam, SV Desert dingo and SV CanFam, respectively. If the SNV and SV Desert dingo X chromosome data are excluded the r^2 of these regressions increases to 0.67 and 0.54, respectively.

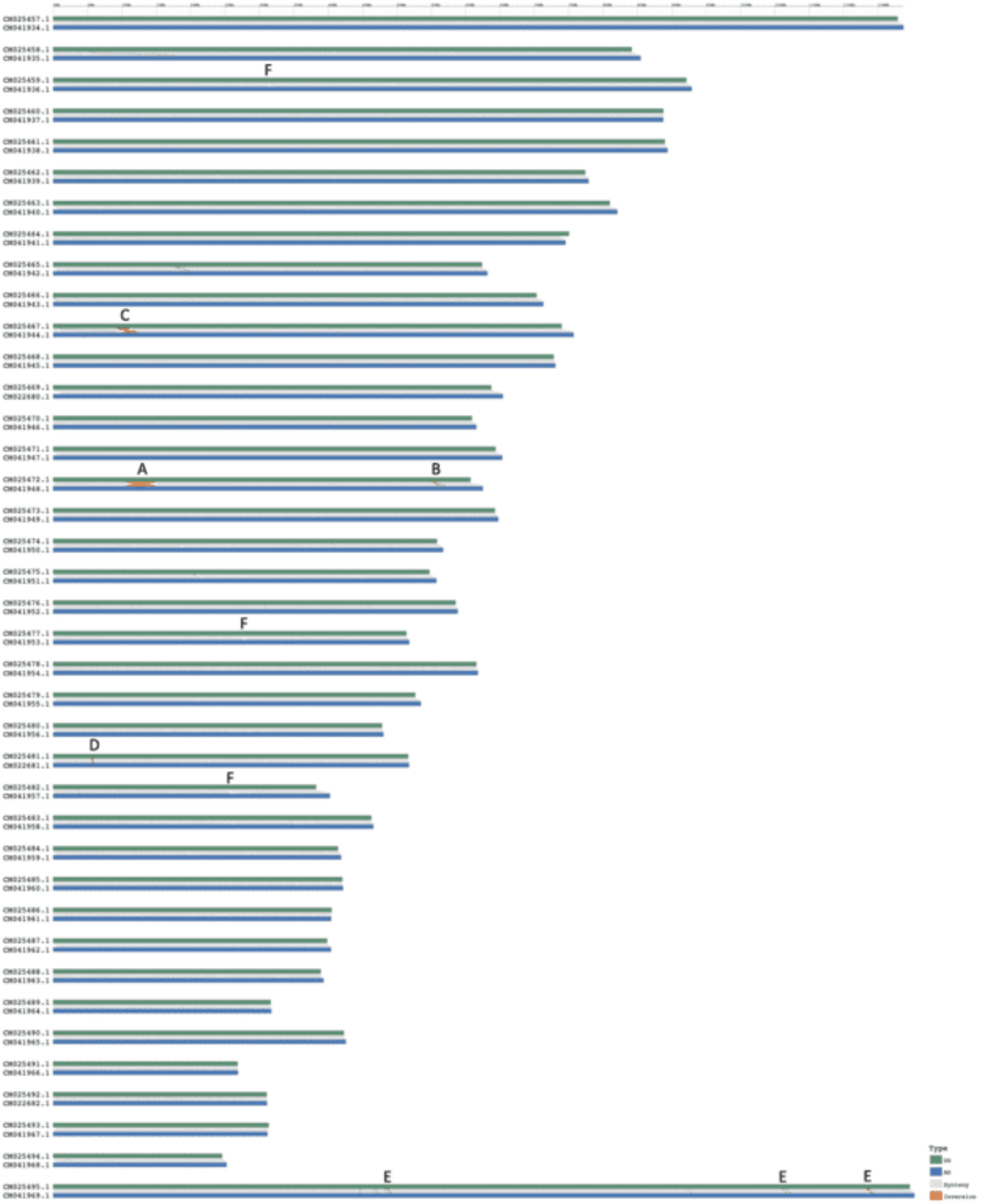

Supplementary Figure 5: Synteny analyses

Supplementary Figure 5 Legend: Synteny plot of Alpine dingo Cooinda (AD) in blue against Desert dingo Sandy in orange (DD). A. Shows the 3.45Mb rearrangement on Chromosome 16. B. Shows the complex rearrangement between 55-57 Mb downstream on Chromosome 16. C Smaller inversion on Chromosome 11. D. Small inversion on

Chromosome 25. E. Multiple possible small inversions on X chromosome. Other smaller rearrangements are possible. F. Possible duplication like events.

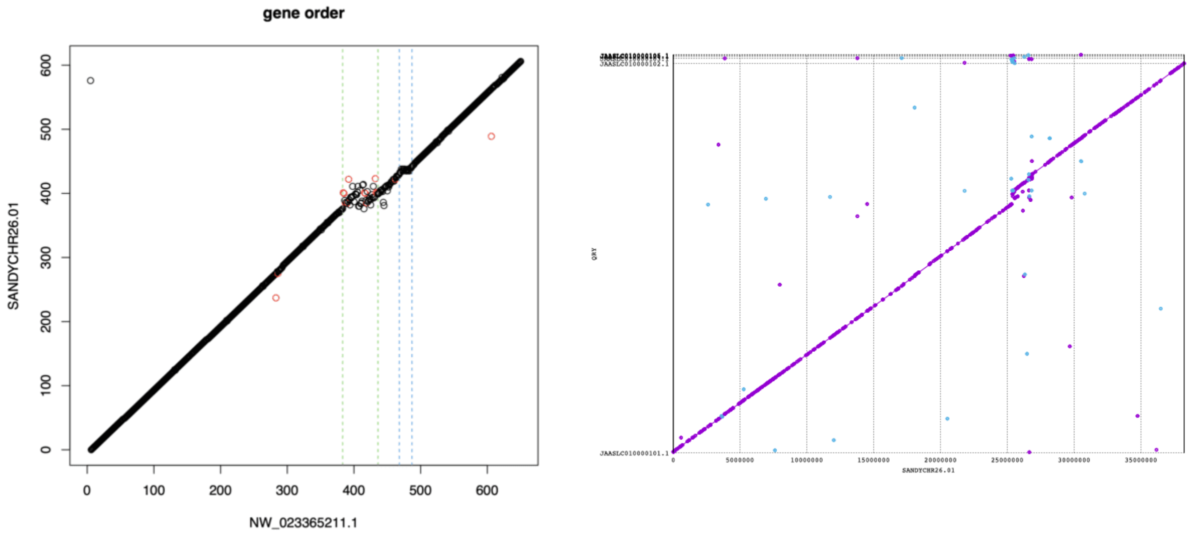

Supplementary Figure 6: Gene order plot comparing Chromosome 26 for Cooinda the Alpine Dingo (X-axis) and Desert Dingo Sandy (Y-axis) using GenomeSym (left) and MUMmer (right).

Supplementary Figure 6 Legend: In GeMoMa plot (left) the green and the blue dashed lines indicate the two structural events on chromosome 26 of Cooinda. The same region is shown using MUMmer (right).

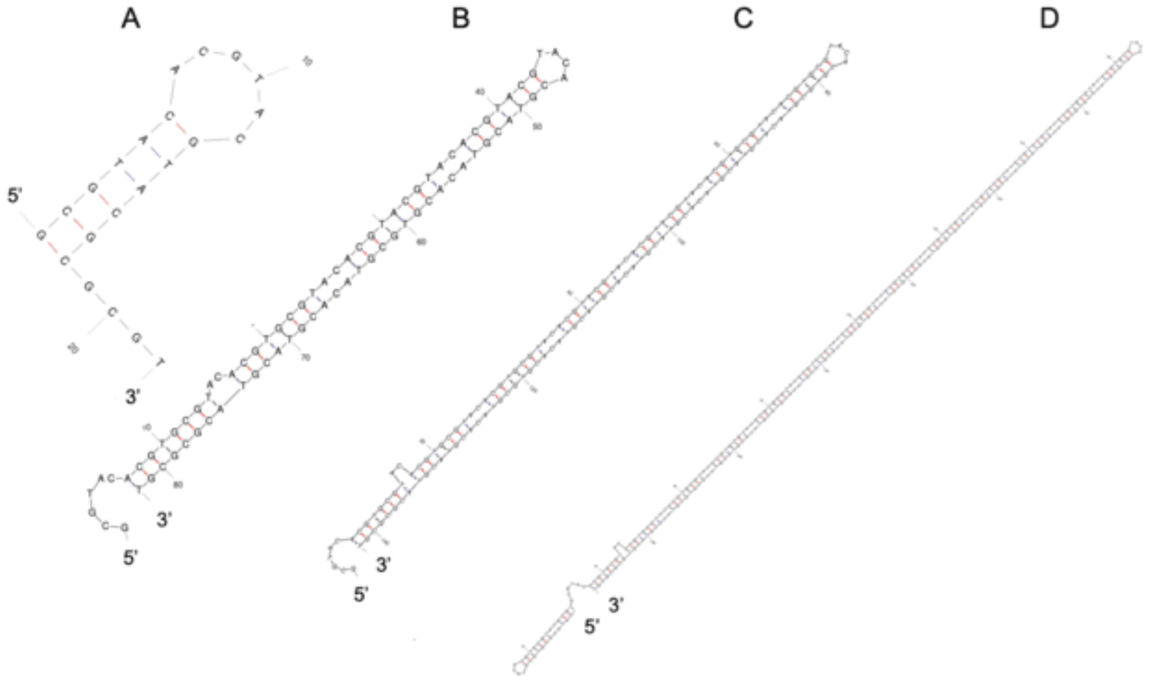

Supplementary Figure 7: Possible folding of 10bp repeats in D-loop region

S Supplementary Figure 7 Legend: (A) 1 repeat, ΔG=-4.68, (B) 7 repeats ΔG=-29.07, (C) 13 repeats ΔG=-48.21, (D) 28 repeats ΔG=-97.71.

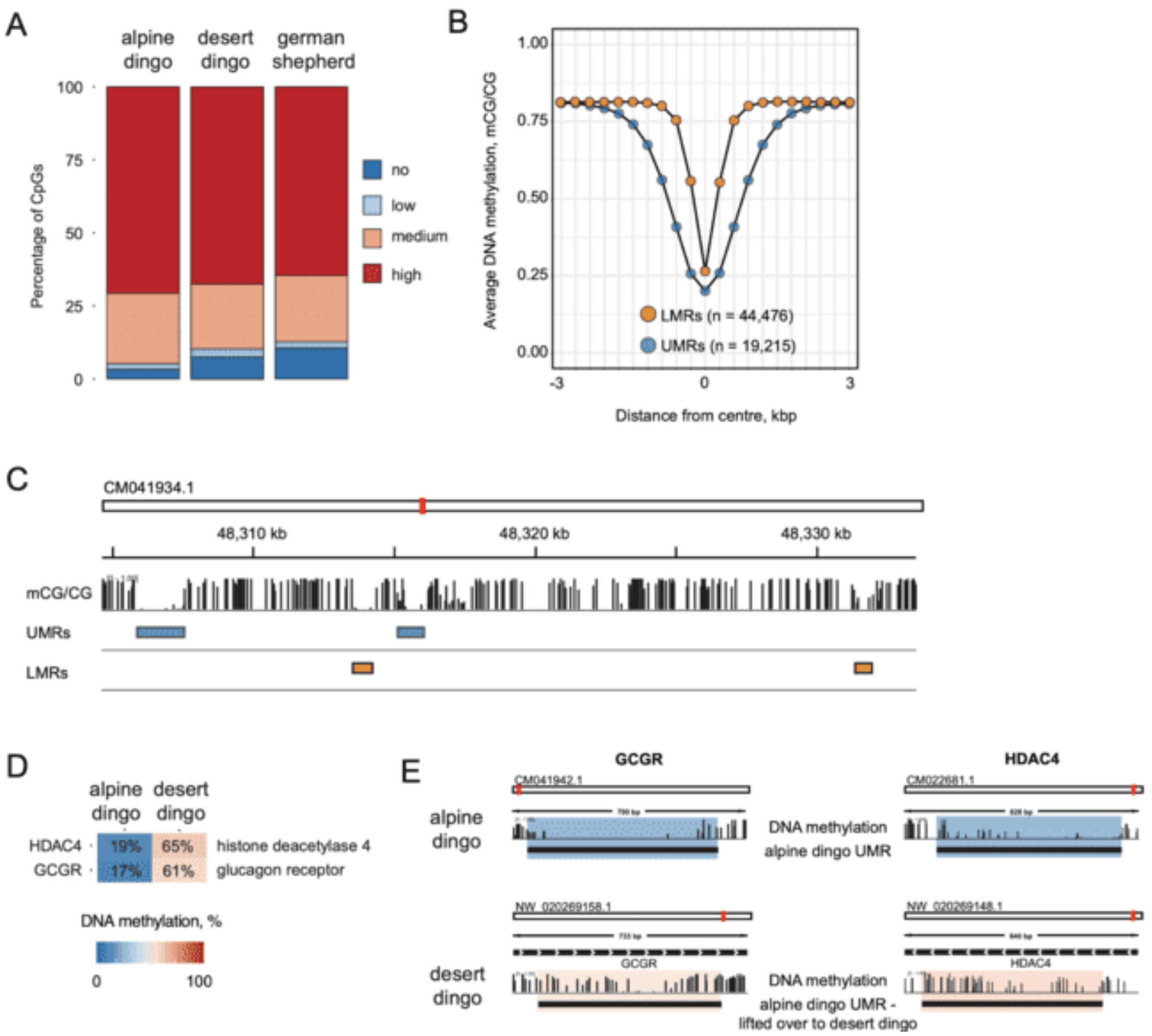

Supplementary Figure 8: DNA methylation profiling of alpine dingo Cooinda's whole blood

Supplementary Figure 8 Legend: (A) Percentage of CpG sites with different levels of methylation. High, 80-100%; medium, 20-80%; low, >0-20%; no, 0%. **(B)** Average DNA methylation profiles of hypomethylated regions into CpG-rich unmethylated regions (UMRs) and CpG-poor low-methylated regions (LMRs). **(C)** Integrative Genomics Viewer **(**IGV) browser track depicting DNA methylation profile and putative regulatory elements (UMRs and LMRs). **(D)** Heatmap depicting average DNA methylation at hypomethylated UMRs in the alpine dingo genome, which are more than 50% methylated in the desert dingo genome. **(E)** IGV browser track depicting hypomethylated UMRs within GCGR and HDAC4 genes in the alpine dingo genome, which are hypermethylated in the desert dingo genome.

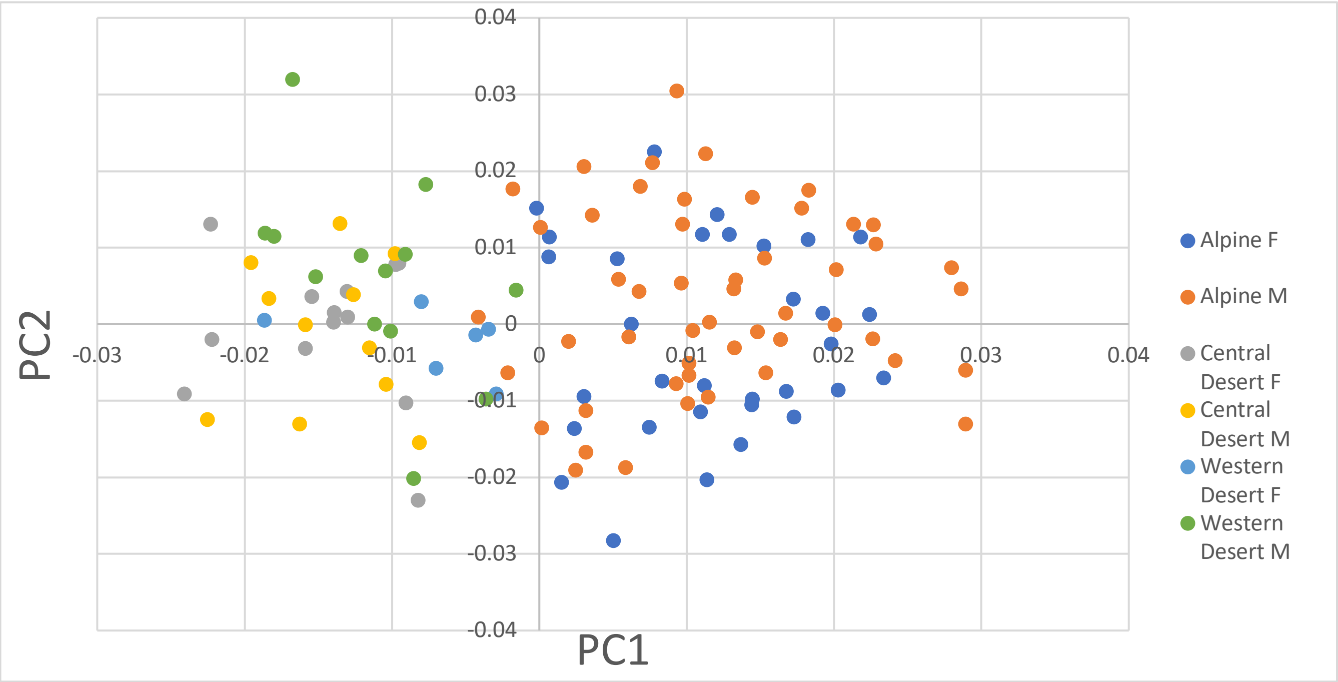

Supplementary Figure 9

Supplementary Figure 9 Legend: Scatterplot of PC1 and PC2 values for sexed dingo specimens. The distribution of greater PC2 values slightly favors males in all populations except for Central Desert, which is a very gracile population with relatively minimal differences between the cranial morphology of different sexes. In general, however, the difference in PC2 between males and females in any population is very marginal and neither greater nor lesser values are particularly strongly associated with either sex.

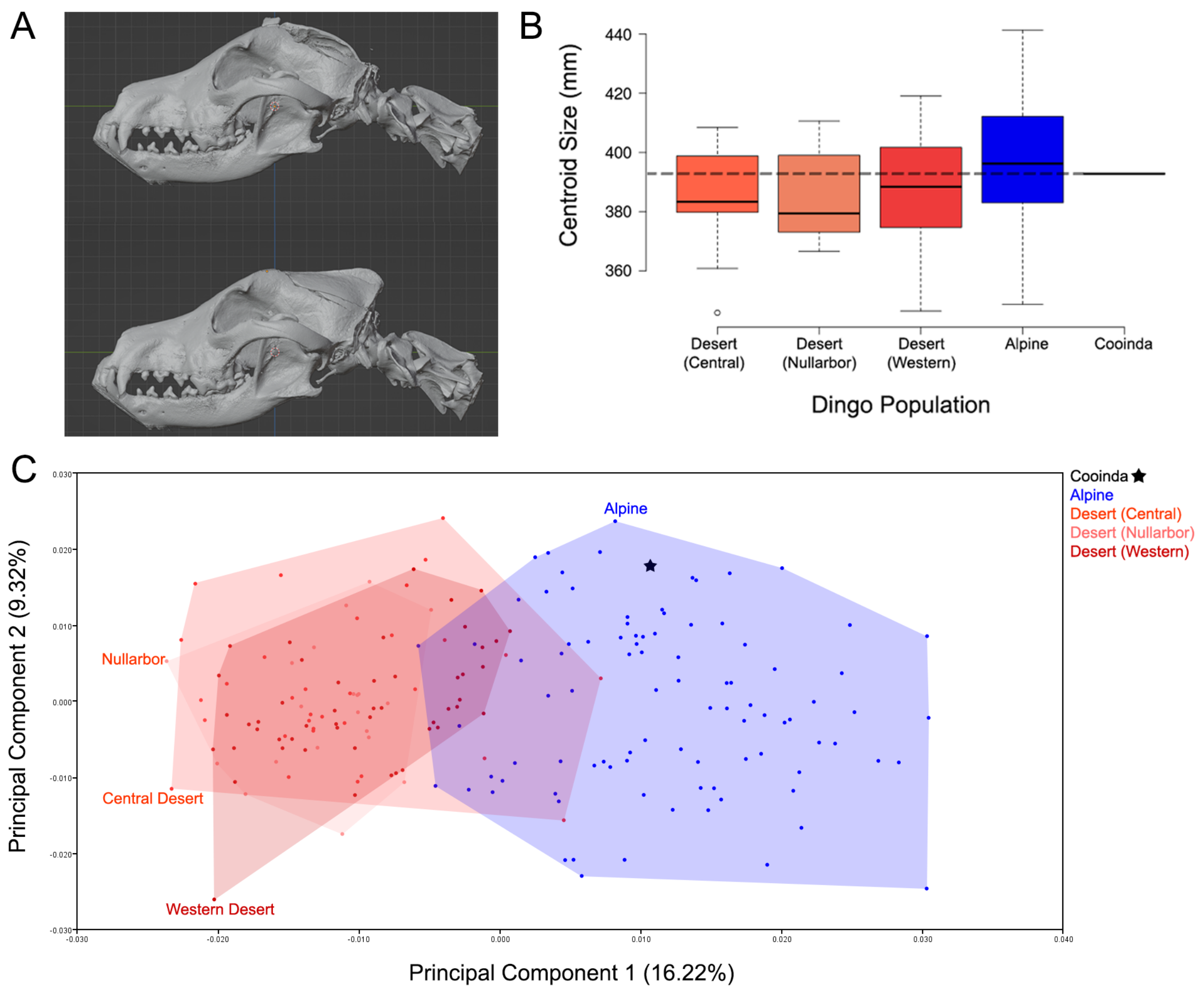

Supplementary Figure 10: Cooinda's cranial morphology

Supplementary Figure 10 Legend: (A) Cranium before (upper) and after (lower) cranial reconstruction (lower). This was required because the brain was removed immediately after death, which caused some damage to the braincase. (B) Cranium size. Cooinda's cranium is larger than the median size reported for Desert dingoes, in line with Alpine dingoes in general, although this difference is not major and there is heavy overlap between the two regions. Her centroid size (392.80mm) is slightly below the pooled Alpine mean (396.49mm) and median (396.23mm), but well below the mean (403.26mm) and median (403.64mm) for Alpine males specifically, which make up a majority of the sample (male n = 50; female n = 33; sex unknown n = 9). Alpine dingoes, as with all regional dingo populations, exhibit significant sexual dimorphism in centroid size with males being on average 4.20% larger

[65]. (C) Principal component ordination of allometric residuals. The residuals of a regression of shape against log centroid size were plotted to further explore the role of size (allometry) in overall form. This revealed that the separation of Alpine and Desert populations, and Cooinda’s position within the former, remains essentially identical to their original distribution s (Fig. 5a) when the size-related allometric component of form is removed from consideration.

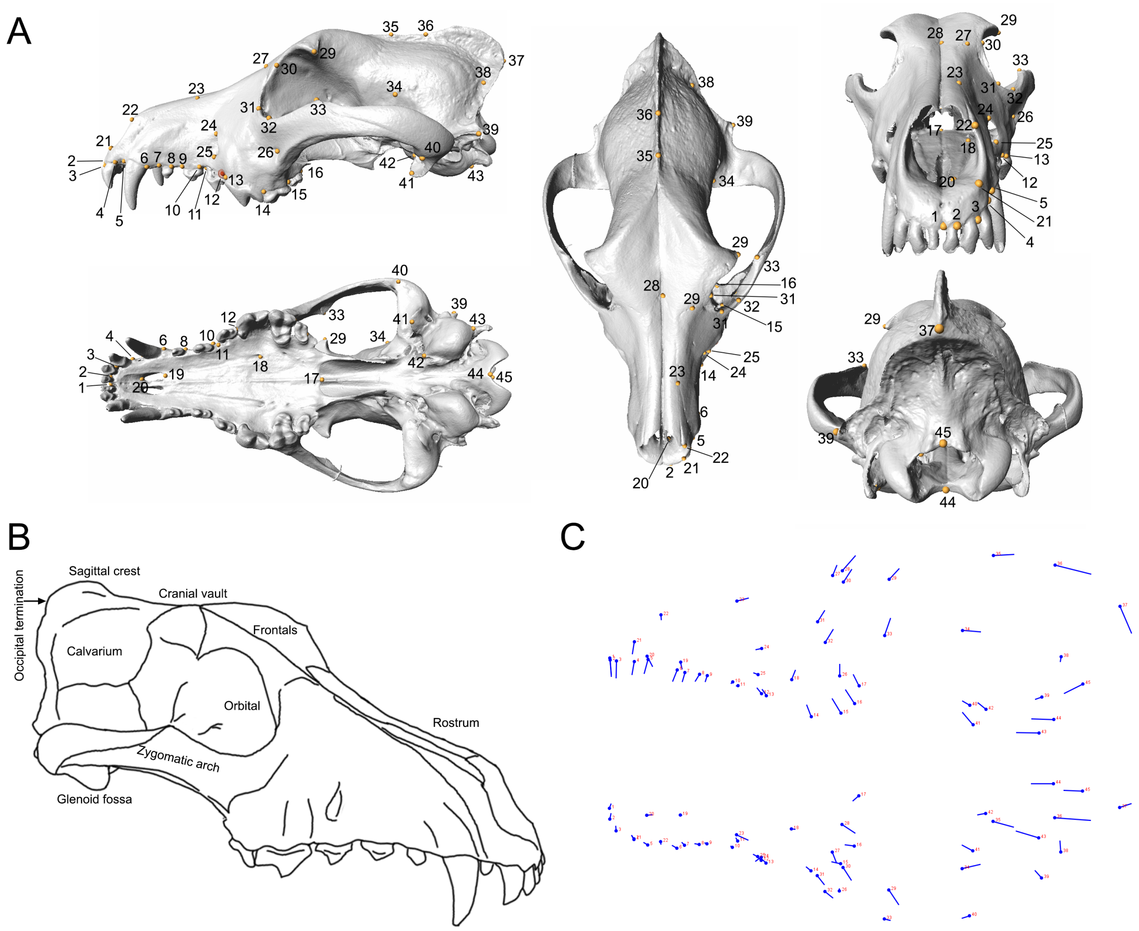

Supplementary Figure 11

Supplementary Figure 11 Legend: (A) Landmarks used in this study. (B) diagram of canid skull with basic anatomical features and regions referred to in-text. (C) Lollipop figures illustrating change in landmark positions along PC2 in lateral (upper) and dorsal (lower) views. The lollipop “head” represents the mean position, and the end of the “stick” represents its position with the highest PC1 score.

Supplementary Table 1. Alpine dingo SNVs and SVs summary by chromosome.

**Chromosome**	**Alpine dingo SNV count vs**		**Alpine dingo SV count vs**		**Chromosome bp**
	**Desert dingo**	**CanFam4.0**	**Desert dingo**	**CanFam4.0**	
**1**	77926	195865	1370	3086	123706252
**2**	55068	134823	1192	2074	85446638
**3**	63384	162076	939	2552	92885137
**4**	53989	145400	1001	2287	88750986
**5**	57509	137691	981	2181	89373516
**6**	49987	114964	1067	2510	77892171
**7**	61659	120334	1132	2074	82078758
**8**	51280	132310	1038	2138	74537974
**9**	48343	87955	1027	1566	63158580
**10**	42060	106101	754	1477	71288900
**11**	54954	119566	1100	1950	75713417
**12**	80643	119102	1661	2123	73055177
**13**	67246	96899	1076	1411	65408697
**14**	44691	97750	899	1913	61550799
**15**	56205	93452	1023	1627	65319253
**16**	51884	99759	1176	2021	62499104
**17**	35216	114685	754	1783	64752584
**18**	50255	93879	1160	1536	56713732
**19**	54419	94065	988	1311	55746904
**20**	40988	86977	703	1446	58849343
**21**	41147	96550	691	1293	51792689
**22**	33737	94950	636	1604	61776113
**23**	44240	87440	794	1274	53490029
**24**	32824	81112	491	1037	48051091
**25**	43100	97505	739	1413	51754766
**26**	38604	91768	757	1406	40258947
**27**	52772	76375	1075	1447	46564242
**28**	29616	81846	517	984	41881905
**29**	29642	75460	507	1121	42159209
**30**	22298	69332	486	1074	40431870
**31**	41002	82966	628	1015	40406310

**32**	26587	83791	614	1610	39322259
**33**	33909	57901	692	977	31731374
**34**	35988	89665	567	1843	42557588
**35**	24494	56238	383	683	26863133
**36**	19944	53165	393	796	31103624
**37**	31481	49270	789	935	31168689
**38**	26115	58654	416	780	25215458
**X**	24584	102071	582	2166	125292608

Supplementary Table 2. Distance matrix table showing SNVs above diagonal and Indels below. All possible pairwise alignments were generated using MUMmer4 [44] (v4.0.0 beta 2) and SNVs/indels numbers calculated using MUMmer4 'show-snp' script.

	**Desert**	**Alpine**	**Basenji1 (China)**	**Basenji2 (Wags)**	**GSD1 (Nala)**	**GSD2 (Mischa)**	**Labrador**	**Boxer**	**Great Dane**	**Greenland Wolf**
**Desert**	-	1934204	4379273	4058304	4157347	4099899	4266975	3956320	3858069	5039138
**Alpine**	3525802	-	4351866	4048746	4125867	4061800	4219881	3939675	3802100	4696525
**Basenji1**	6813866	6946862	-	2199194	3893739	3855744	3922731	3700555	3605316	5155027
**Basenji2**	6482039	6567647	4372616	-	3515180	3471928	3686649	3375257	3246911	4949577
**GSD1**	6290364	6362924	6237235	5742582	-	2064119	3477794	3101130	3007546	5078984
**GSD2**	6229282	6301226	6186934	5663021	3396958	-	3426212	3072348	2990169	5049776
**Labrador**	7072684	7122926	6758598	6529100	6029553	5975640	-	3174798	3162230	5252186
**Boxer**	6124985	6229361	6004983	5678411	5061694	5025968	5816010	-	2773431	4947075
**Great Dane**	6601081	6693070	6455860	6160610	5582788	5578574	6440463	5255792	-	4776954
**Greenland Wolf**	7273469	6925872	7606227	7343748	7339762	7266186	8130863	7190906	7642878	-

GSD is German shepherd dog

Supplementary Table 3. Mean and median PC2 scores for different sexes from dingo populations

.

	PC2 Mean	PC2 Median
Alpine F	-0.00203	-0.00481572
Alpine M	0.003143	0.001463463
Central Desert F	-0.00062	0.000931911
Central Desert M	-0.00131	-3.97883E-05
Western Desert F	-0.00226	-0.001064826
Western Desert M	0.00602	0.006917365

Supplementary Table 4. List and description of cranial landmarks used in this study. After Koungoulos [65].

Landmark #	*Description*
1	*Between left and right first incisors.*
2	*Between first and second incisor.*
3	*Between second and third incisor.*
4	*Posterior corner of third incisor alveolus.*
5	*Anterior corner of canine alveolus.*
6	*Posterior corner of canine alveolus.*
7	*Anterior corner of first premolar alveolus.*
8	*Posterior corner of first premolar alveolus.*
9	*Anterior corner of second premolar alveolus.*
10	*Posterior corner of second premolar alveolus.*
11	*Anterior corner of third premolar alveolus.*
12	*Posterior corner of third premolar alveolus.*
13	*Anterior corner of fourth premolar (carnassial) alveolus.*
14	*Posterior corner of fourth premolar (carnassial)/Anterior corner of first molar alveoli.*
15	*Posterior corner of first molar (carnassial)/Anterior corner of second molar alveoli.*
16	*Posterior edge of second molar alveolus.*
17	*Staphylion at edge of palate and choanal region.*
18	*Greater palatal foramen.*
19	*Proximal end of palatine fissure.*
20	*Distal end of palatine fissure.*
21	*Lower corner of distal incisive bone.*
22	*Upper corner of distal incisive bone.*
23	*Intersection of incisive, maxilla and nasal bones.*
24	*Upper end of infraorbital foramen ridge.*
25	*Lower end of infraorbital foramen ridge.*
26	*Lower limit of intersection of maxilla and zygomatic bones.*
27	*Upper limit of intersection of maxilla and frontal bones.*
28	*Upper limit of intersection of nasal and frontal bone.*
29	*Furthest extent of zygomatic process of frontal bone.*
30	*First "corner" of orbital rim.*
31	*Second "corner" of orbital rim.*
32	*Third "corner" of orbital rim.*
33	*Fourth "corner" of orbital rim.*
34	*Intersection of frontal, parietal and temporal bones.*
35	*Intersection of cranial midline with frontal-temporal boundary suture (bregma).*
36	*Intersection of the parietal and occipital bones on the sagittal crest.*
37	*Most proximal extent/tip of occipital protuberance (occiput).*
38	*Intersection of nuchal crest with parietal-temporal boundary suture.*
39	*Lower flare of nuchal crest.*
40	*Underside of mandibular fossa, where zygomatic process begins.*

41	*Underside of retroarticular process.*
42	*Anterior corner of auditory bulla.*
43	*Posterior corner of auditory bulla.*
44	*Lower central rim of foramen magnum, between occipital condyles.*
45	*Upper central rim of foramen magnum, between occipital condyles.*
